# Health care provided to recent asylum-seeking and non-asylum-seeking pediatric patients in 2016 and 2017 at a Swiss tertiary hospital - a retrospective study

**DOI:** 10.1186/s12889-020-10082-z

**Published:** 2021-01-07

**Authors:** Julia Brandenberger, Christian Pohl, Florian Vogt, Thorkild Tylleskär, Nicole Ritz

**Affiliations:** 1 University of Basel Children’s Hospital, Migrant Health Service, Spitalstrasse 33, 4056 Basel, Switzerland; 2 Pediatric Emergency Department, University Children’s Hospital, Inselspital, University of Bern, Bern, Switzerland; 3 Neonatal Intensive Care Unit, Perth Children’s and Kind Edward Memorial Hospitals, Perth, Australia; 4 Unit of NTDs, Department of Clinical Sciences, Institute of Tropical Medicine, Antwerp, Belgium; 5 Centre for International Health, University of Bergen, Bergen, Norway; 6 University of Basel Children’s Hospital, Pediatric Infectious Disease and Vaccinology, Basel, Switzerland; 7 Department of Pediatrics, Royal Children’s Hospital Melbourne, University of Melbourne, Parkville, Australia

**Keywords:** Migrant and refugee health, Children, Use of health care, Migration patterns, Europe, health care delivery

## Abstract

**Background:**

Asylum-seeking children represent an increasing and vulnerable group of patients whose health needs are largely unmet. Data on the health care provision to asylum-seeking children in European contexts is scarce. In this study we compare the health care provided to recent asylum-seeking and non-asylum-seeking children at a Swiss tertiary hospital.

**Methods:**

We performed a cross-sectional retrospective study in a pediatric tertiary care hospital in Basel, Switzerland. All patients and visits from January 2016 to December 2017 were identified, using administrative and medical electronic health records. The asylum-seeking status was systematically assessed and the patients were allocated accordingly in the two study groups.

**Results:**

A total of 202,316 visits by 55,789 patients were included, of which asylum-seeking patients accounted for 1674 (1%) visits by 439 (1%) individuals. The emergency department recorded the highest number of visits in both groups with a lower proportion in asylum-seeking compared to non-asylum-seeking children: 19% (317/1674) and 32% (64,315/200,642) respectively. The median number of visits per patient was 1 (IQR 1–2) in the asylum-seeking and 2 (IQR 1–4) in the non-asylum-seeking children. Hospital admissions were more common in asylum-seeking compared to non-asylum-seeking patients with 11% (184/1674) and 7% (14,692/200,642). Frequent visits (> 15 visits per patient) accounted for 48% (807/1674) of total visits in asylum-seeking and 25% (49,886/200,642) of total visits in non-asylum-seeking patients.

**Conclusions:**

Hospital visits by asylum-seeking children represented a small proportion of all visits. The emergency department had the highest number of visits in all patients but was less frequently used by asylum-seeking children. Frequent care suggests that asylum-seeking patients also present with more complex diseases. Further studies are needed, focusing on asylum-seeking children with medical complexity.

## Background

Globally, an estimated 68.5 million individuals and 35.5 million children were forcibly displaced in 2017 [1]. This number has reached a historical record high, with child refugees having increased by 21% in the past decade [2]. In Europe, 209,756 children have claimed asylum in 2017, of which 6595 lodged their application in Switzerland [3]. In the same year, a further 16,350 children were registered as temporarily accepted refugees in Switzerland [4].

The remarkable increase of asylum-seeking children and adults arriving in Europe may challenge the health care systems. Thus, concerns about the quality of medical care provided to asylum-seekers have led to several international reports and action plans [5, 6]. These highlight that asylum-seeking children are a particularly vulnerable group whose health needs are largely unmet [6, 7].

The majority of available data on health care provision and needs in asylum-seekers focuses on adults or originates from the late 1990s. Current asylum-seeking populations in Europe are, however, distinctly different compared to 1990s when the majority of asylum-seekers originated from the Balkan states and the proportion of children was reaching only 10% [8–12]. In recent years, however, the majority of asylum-seekers originate from the horn of Africa and middle Eastern countries and on average 30% are children and adolescents [13].

A systematic literature review on the health of migrant children in Switzerland including evidence until 2011 concluded that migrant children had important differences in health needs compared to their local peers reflected in higher hospital admission rates, intensive care admissions, dental care and mental health consultations [14]. In contrast to this, a recent study of asylum-seeking hospitalized children showed that a large proportion was admitted with infections similar to those prevalent in the local population. However, a direct comparison between the groups was not done [15]. Only one recent study from Germany included health care delivery data from both asylum-seeking and non-asylum seeking children [16]. The study showed that asylum-seeking children were more frequently admitted for diseases with the potential for outpatient care, when detected early.

In summary, there is a knowledge gap on recent health data of asylum-seeking children in comparison with non-asylum-seeking children. This absence of information has been highlighted as a research priority by several international organizations including the World Health Organization (WHO) and the International Society for Social Pediatrics and Child Health (ISSOP) [5, 17, 18].

The aim of this study was therefore to fill an important knowledge gap by comparing health visits from asylum-seeking and non-asylum-seeking children to analyze and understand differences in their health needs.

## Methods

### Study area

The study was done at the University Children’s Hospital Basel (UKBB). Located in Switzerland on the border to France and Germany, the University Children’s Hospital Basel delivers health care to a multicultural population, aged between 0 and 16 years ans exceptionally to older patients. The hospital is part of the Swiss hospitals for equity program [19] and the only tertiary pediatric health care provider for two regions in North-West Switzerland with on average 200,000 consultations per year. Since March 2019, Switzerland is divided into six asylum regions. Basel has the only federal asylum center (FAC) with processing facilities within the asylum region. It is run by the Swiss state secretary of migration. All children lodging at the federal asylum center in need of urgent medical care are referred to the university children’s hospital. In addition, asylum-seeking families relocated to apartments within the region can spontaneously present themselves at the hospital.

### Study population

In this cross-sectional study data of all visits at the University Children’s Hospital Basel occurring between the two previous years, namely from 1st Jan 2016 and 31st Dec 2017 were extracted from the administrative electronic health records. The asylum-seeking status was systematically assessed and recorded at our institution for all patients. Patients were registered as asylum-seeking if any of the following conditions were met: (i) referred from one of the reception and processing centers run by the State Secretary for Migration; (ii) referral sheet stating that the patient is asylum-seeking; (iii) asylum-seeking identity card, which is routinely issued to all individuals lodging an asylum request in Switzerland. As the health profile of asylum-seeking persons is assimilating to the local population in many health related aspects like infectious diseases or risk behavior [20]. To prevent the dilution of important differences, only recently arrived asylum-seeking patients were included. Children that had visits recorded 1 year or longer before the study period (i.e. before 1st January 2015) were excluded from the current analysis.

### Data collection and analysis

Data extraction for all identified patients was done in 2018 and 2019 using administrative and medical electronic health records for the following variables: number of visits per group as primary outcome and asylum status, nationality, age, gender, date of visit, department visited, time of visit (office hour visit defined as 7:00–5:59 pm), hospital admission or outpatient treatment. Extracted data was transferred to a Redcap-database (Vanderbilt University/IC 6.9.4). Data cleaning and quality control tests were performed.

STATA (Stata/IC 13.12013) was used for the statistical analysis as for the generation of graphs. The statistical analysis was mainly descriptive. Inferential statistics were used to analyze key differences between the two groups, using the two sample Chi-square test. Confidence intervals were provided to describe the precision around the summary statistic using a confidence level of 95%. To provide information about the completeness of the dataset, records with missing data were not excluded from analysis but reported as such.

### Ethics

The study was approved by the Ethics committee of North-West Switzerland (EKNZ 2017–01585). Informed consent was not required as per EKNZ as not deemed feasible for the analysis of the large dataset. No further administrative permissions were required to access the raw data for the study.

## Results

### Primary outcome

**Fig. 1:**
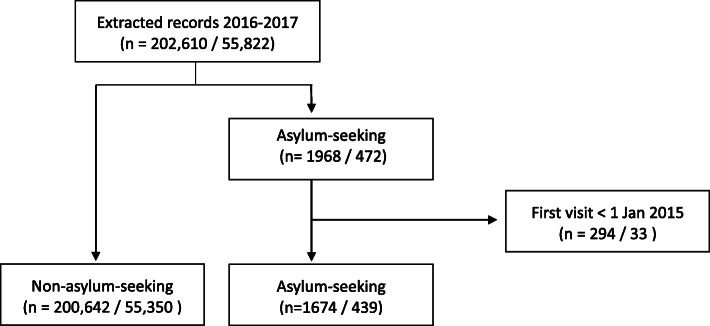
Flow-diagram showing the process of inclusion of the study population. The flow-diagram was created by the first author of this study

The final study population included 202,316 visits by 55,789 patients of which 1674 (1%) visits by 439 (1%) patients were in asylum-seeking and 200,642 (99%) visits by 55,350 (99%) patients were in non-asylum-seeking children (Fig. 1). A total of 294 visits by 33 asylum-seeking patients were excluded as these had records of visits prior to 1st Jan 2015 (Table 1).

**Table 1:** Baseline characteristics, nationality and most visited departments by asylum-seeking and non-asylum-seeking patients 2016–2017 at University Children’s Hospital Basel

	Asylum-seeking				Non-asylum-seeking				
Characteristics	Visits ***n*** = 1674		Patients ***n*** = 439		Visits ***n*** = 200,642			Patients ***n*** = 55,350	
	N	%	N	%	N		%	N	%
**Age groups:**									
0–2	646	39	96	22	45,478		23	13,297	24
3–14	575	34	152	34	117,546		58	32,819	59
15–17	421	25	171	39	25,736		13	5938	11
> 17	32	2	20	5	11,882		6	3296	6
Total 2016	812	49	243	55	100,842		50	36,560	66
Total 2017	862	51	243	55	99,800		50	36,767	66
Male gender	1172	70	307	70	110,567		55	29,520	53
**Most frequent nationalities:**									
Syria	442	26	41	9	Switzerland	124,714	62	35,381	64
Eritrea	210	13	60	14	Germany	12,961	6	3853	7
Afghanistan	192	11	58	13	Turkey	9310	5	2080	4
Algeria	182	11	4	1	Italy	7292	4	1984	4
Armenia	157	9	4	1	Kosovo	5368	3	1325	2
Other	371	22	154	35	Other	40,973	20	10,714	19
Missing Data^a^	120	7	118	27	Missing Data	24	0	13	0
Outpatient visits	1490	89	ns	ns	185,950		93	ns	ns
Office hour visits (7.00 am – 5.59 pm)	1505	90	ns	ns	167,901		84	ns	ns
**Most visited outpatient departments:**									
Emergency	317	19	ns	ns	Emergency	64,315	32	ns	ns
Exercise therapy	200	12	ns	ns	Surgery	18,507	9	ns	ns
Radiology	173	10	ns	ns	Orthopedics	16,225	8	ns	ns
Occupational therapy	144	9	ns	ns	Ear, nose, throat	15,775	8	ns	ns
Haemato-oncology	141	8	ns	ns	Neurology	10,338	5	ns	ns

*ns* Not specified

^a^ Missing data nationality asylum-seeking: in 95% of cases patients only seen for radiological examination as part of a State Secretary of Migration age estimation-programme

### Nationality

**Fig. 2:**
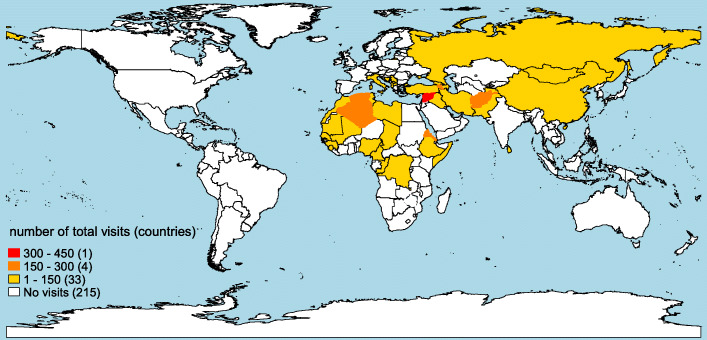
Number of visits by asylum-seeking patients per country. The map was created by the first author of this study

Asylum-seeking patients originated from 38 countries and the most frequent nationalities were Eritrea (14%), Afghanistan (13%), and Syria (9%). In the non-asylum-seeking patients 141 countries were noted with the majority from Switzerland (64%), followed by Germany (7%), Turkey and Italy (4% each) (Table 1; Fig. 2).

### Age and sex

**Fig. 3:**
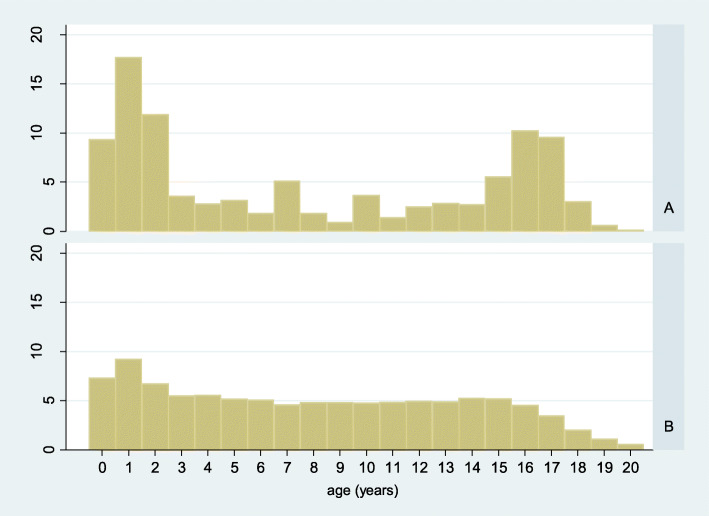
Age distribution of visits of asylum-seeking children (*n* = 1674; panel **a**) and non-asylum-seeking children (*n* = 200,642; panel **b**) in the years 2016 and 2017. Some few patients aged above 20 years are not depicted. The graph was created by the first author of this study

The median age was higher in the asylum-seeking compared to the non-asylum seeking patients: 13 (IQR 3–16) years and 7 years (IQR 2–12), respectively. In both groups, visits from children < 3 years of age were most frequent with 39% (646/1674) and 23% (45,478/200,642) in the asylum-seeking and non-asylum-seeking patients; with the proportion being significantly higher in asylum-seeking patients (p-value < 0.001, 95% CI difference in proportion: 0.14–0.18) (Fig. 3). A bimodal age-distribution was seen in the asylum-seeking patients with a second peak in adolescents aged 15 to 17 years. Visits from adolescents represented 25% (421/1674) in the asylum-seeking and 13% (25,736 / 200,642) in the non-asylum-seeking group (*p* < 0.001, 95% CI difference in proportion: 0.1–0.14).

There was a predominance of male patients among the asylum-seeking compared to the non-asylum-seeking patients, 70% versus 55% of visits (*p* < 0.001, 95% CI difference in proportion: 0.13–0.17). This difference was highest in the adolescent group with 87% (366/421) of visits by males in the asylum-seeking and 50% (12,922/25,736) in the non-asylum-seeking group, respectively. The sex distribution in children < 3 years was similar in both groups; 54% males (352/646) in asylum-seeking and 56% (25,347/45,478) in non-asylum-seeking patients, respectively.

### Visits stratified by departments

Outpatient visits were most frequent in both groups with 89% (1490/1674) in the asylum-seeking and 93% (185,950/200,642) in the non-asylum-seeking patients, respectively. The emergency department was most frequently visited in both groups with a significantly lower proportion in the asylum-seeking patients with 19% (317/1674, CI: 0.15–0.23) compared 32% (64,315/200,642; CI: 0.32–0.32) in the non-asylum-seeking patients (*p* < 0.001, 95% CI of difference in proportion: -0.17− -0.09).

The proportion of hospital admissions was higher in asylum-seeking patients with 11% (184/1674) compared to 7% (14,692/200,642) in the non-asylum-seeking children (*p* < 0.00, 95% CI difference in proportion: 0.02–0.06). Asylum-seeking patients were most frequently admitted to the pediatric department (36% (67/184)) and non-asylum-seeking patients to the surgical/orthopedic department (40% (5857/ 14,692)). Hospital admissions to psychiatry were infrequent in both groups with 4% (7/184) in asylum-seeking and 2% (282/14,692) in non-asylum-seeking patients.

### Frequency of health care visits

**Fig. 4:**
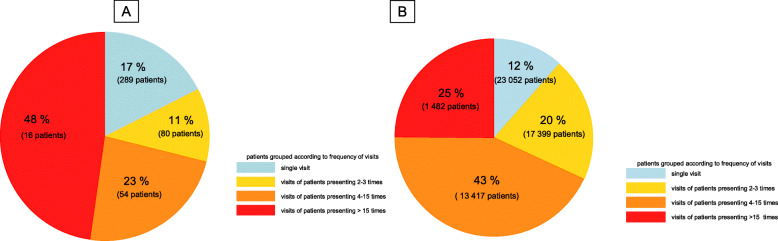
Pie charts depicting frequency of visits and proportions of total visits by **a** asylum-seeking (*n* = 439 with 1674 visits) and **b** non-asylum-seeking children (*n* = 55,350 with 200,642 visits) in 2016 and 2017. The graph was created by the first author of this study

The median number of visits per patient was 1 (IQR 1–2; range 1–179) in the asylum-seeking and 2 (IQR 1–4; range 1–221) in the non-asylum-seeking children. The frequency of visits per patient was < 4 times in 84% (369/439) and 72% (40,451/55,350), of the asylum-seeking and non-asylum-seeking children, respectively. When analyzing patients with frequent visits (> 15 visits per patient), these occurred in 4% (16/439) of the asylum-seeking and in 3% (1482/55350) of the non-asylum-seeking patients. Frequent visits accounted for 48% (807/1674) and 25% (49,886/200,642) of the total visits in the asylum-seeking and non-asylum-seeking patients, respectively (Fig. 4).

## Discussion

This is the first comprehensive analysis of hospital visits comparing pediatric asylum-seeking with non-asylum-seeking patients in Europe. Overall, the number of visits by asylum-seeking patients was small but the comparison to visits of non-asylum-seeking patients showed important differences.

Asylum-seeking patients originated from a considerable number of countries with Afghanistan, Eritrea and Syria being the most frequent countries of origin in the period studied. Compared to our previous analysis of asylum-seeking patients in 2015 the main difference is that Syrian patients have become more frequent. This shift is a result of current migration patterns in which Syrians represented 54% of the total number of recorded arrivals in Europe in 2015 and 2016. In 2018, 2.7 million Syrian refugee children were living outside of Syria [21]. Due to the severity and complexity of the armed conflict, Syria has shifted from a temporary to a permanent country of origin of refugees. This results in a continuously decreasing health status of Syrian citizens as also demonstrated that by a recent study in which only 64% had access to general pediatric care, 28% had up-to-date vaccination status and 16% access to healthy nutrition [22]. The Syrian context is contrasted by Afghanistan, which has been one of the top 20 countries of origin of refugees since the 1980s [1]. These changing trends in nationalities, contexts and demographics of the asylum-seeking population influence the health needs of asylum-seekers and highlight the need for host countries to continuously monitor their practice of health provision.

A substantial number of visits by asylum-seeking patients were by male adolescents. This is an important patient group reflecting the current age and sex distribution among refugees in Europe. In 2017, 82% of the first-time asylum seekers were less than 35 years old and 75% of the 14 to17 years old asylum-seekers were male, many being unaccompanied minor refugees [23]. The frequency of this age group in hospital visits may be a surrogate for poor health or limited access to health care in this group of refugees. The Unaccompanied Refugee Minors Program of the United States showed that and that long-term health care remained challenging in this group [24]. The results of our study show that most visits in this age group were in somatic departments and relatively few admissions to the psychiatric department were noted. This is somewhat surprising in the light of literature describing the importance of mental health problems in asylum-seeking adolescents [24–29]. One explanation may be cultural differences in expressing mental health needs. Symptoms may appear somatic to health care providers in high-resource countries and underlying mental health problems may have passed undetected [24].

A further important age group in the asylum-seeking patients was children below 3 years of age; however, this was also the case in the non-asylum-seeking patients. In both groups a considerable number of emergency department visits were noted. A similar age distribution in pediatric emergency department visits was seen in other parts of the world. A Californian and a Korean study both showed frequent visits to emergency departments were more common in children aged 1 to 4 years [30, 31]. However, these studies did not detail if asylum-seeking children were included. Interestingly, in our study the asylum-seeking children had a lower proportion of emergency department visits compared to non-asylum-seeking children. This finding contrasts to a recently published study, showing that asylum-seeking children were 5 times more likely to use emergency services [16]. One explanation for the lower proportion in our setting may be that the nurse-led health care system present at Swiss asylum-seeking reception centers which may help to prevent visits to the emergency department, as diseases are detected early. Alternatively, it is possible that asylum-seeking children did not have sufficient access to the emergency department, as health care delivery to migrants includes particular challenges in health care delivery [32].

The generally low proportion of 1% of visits by asylum-seeking patients and the lower proportion of emergency department visits are in line with results from a recent report by the University College London Lancet Commission on Migration [33]. The results underline that public statements in current debates about asylum-seekers disproportionately burdening the health care system are not true for all settings [33]. A study done at an emergency department at the inner city of London also echoed these results, showing that asylum-seekers were only a minority group [34]. Improved access to community-based physicians was described as an option to improve health care and lower the impact of migrants on emergency departments in general [34]. Current health care delivery models to asylum-seekers vary substantially between regions and countries. In our research context, asylum-seeking children are fully covered for all health care visits by the national health insurance. As mentioned in a German study, presentations with ambulatory care sensitive conditions at tertiary health care facilities could be used as an indicator to compare primary care delivery models for asylum-seekers in different regions [16].

One further important finding of our study is that a small proportion of asylum-seeking patients had an outsized number of visits accounting for almost half of the visits. An emerging area of pediatric research focuses on “children with medical complexity”, which typically need frequent health care visits and high financial resources [35]. One likely explication is that asylum-seeking patients presented with serious and inadequately treated medical problems, as their health needs had not sufficiently been addressed in their country of origin and while being on the escape. Once arrived in the host country, they required more intense and prolonged treatment compared to their local peers with the same conditions. Alternatively, the spectrum of disease in asylum-seeking and non-asylum-seeking patients with frequent visits may be different and asylum-seeking patients may suffer from particularly complex or rare diseases [36]. A study analyzing adults with multiple chronic diseases showed that their average annual health care expenditures were three times higher compared to patients without chronic diseases [37]. Despite costs, investment in pediatric patients is generally considered to be cost effective, as it is preventing expensive chronic conditions in adulthood [38]. A third explication for more frequent visits could be that they the asylum-seeking patients had less access to primary care pediatricians, resulting in more frequent presentations at the tertiary health care facility.

This study has several limitations. First, it is a single-center study. The generalizability of the results is therefore limited.

The systematic registration of patients as “asylum-seeking” allowed identifying the health information of this study population. This is considered as strength of the study and described as urgently needed in more settings [39]. However, some asylum-seeking patients might have been missed by administration staff and the number of asylum-seeking patients was potentially underestimated.

This study focused on recent asylum-seekers and therefore excluded visits by asylum-seeking children who visited the hospital before 2015. This helped to prevent the dilution of differences between recent asylum-seekers and non-asylum-seekers. The exclusion of these visits might however limit the results and excludes data from long-term asylum-seeking children. Of note, the proportion of children with non-Swiss nationality in the comparison group with 36% is considerable. Potential differences between Swiss nationals and non-asylum-seekers without Swiss nationality were missed. In addition, migrant children without official documents are not represented in this study.

Another limitation was that the retrospective nature of the study resulted in missing data. Important questions, such as transition countries of asylum-seeking patients before they reached Switzerland could not be included in the analysis as the percentage of missing data was too high. Contacting patients to gather additional information was not deemed feasible due to the size of the dataset. This limited an in-depth analysis of many aspects like analyzing the main diagnosis leading to the visits, the family structure or describing the socio-economic background of the study population, but allowed to include all visits, providing a comprehensive overview of health visits by asylum-seeking patients. To address some of the research questions mentioned in the limitations, further studies are analyzing smaller subgroups of the study population. This allows the comparison of main diagnosis in asylum-seeking and non-asylum-seeking inpatients [40], an in-depth analysis of the subgroup of frequently visiting patients [41] as the investigation of potentially preventable hospital admissions and emergency department visits [42]. Further to this an earlier qualitative study from our institution was done to understand the perspective of the asylum-seeking families on including on aspects for the escape and the quality of care provided at the hospital [43].

Finally, patients which required a change of wards were counted as separate hospital admission. This allowed us to correctly identify all wards where patients were admitted, however, this may have resulted in overestimation of hospital admissions in both groups.

## Conclusions

In summary, hospital visits by asylum-seeking children represented a small proportion of all visits. The emergency department had the highest number of visits in all patients but was less frequently used by asylum-seeking children. Nationalities, age and sex distributions of asylum-seeking patients vary over time and should be considered to identify important specific health needs in asylum-seeking patients. Higher admission rates and a larger proportion of visits from frequently visiting patients suggest that some asylum-seeking children present with more complex diseases. Further studies are needed, focusing on asylum-seeking children with medical complexity.

## Data Availability

The datasets used and/or analysed during the current study are available from the corresponding author on reasonable request.

## Abbreviations

CI: Confidence interval
ED: Emergency department
EKNZ: Ethics committee of North-West Switzerland
FAC: Federal asylum centre
ICD 10: International Classification of Diseases 10
IOM: International Organization for Migration
IQR: Interquartile Range
ISSOP: International Society for Social Pediatrics and Child Health
REDCap: Datamanagement software
SDG: Sustainable Development Goal
STATA: Statistical analysis software
UCL: University College London
UKBB: Universitäts- Kinderspital beider Basel (University Children’s Hospital Basel)
UN: United Nations
UNHCR: United Nations High Commissioner for Refugees
WHO: World Health Organization
